# Palliative care for rural growth and wellbeing: identifying perceived barriers and facilitators in access to palliative care in rural Indiana, USA

**DOI:** 10.1186/s12904-022-00913-8

**Published:** 2022-02-19

**Authors:** Nasreen Lalani, Yun Cai

**Affiliations:** School of Nursing, Purdue University, West Lafayette, Indiana USA

**Keywords:** Palliative care, Access, Rural, Barriers, Facilitators disparities, Healthcare providers

## Abstract

With the growing aging population and high prevalence of chronic illnesses, there is an increasing demand for palliative care. In the US state of Indiana, an estimated 6.3 million people are living with one or more chronic illnesses, a large proportion of them reside in rural areas where there is limited access to palliative care leading to major healthcare inequities and disparities. This study aims to identify common barriers and facilitators to access palliative care services in rural areas of Indiana from the perspectives of healthcare providers including clinicians, educators, and community stakeholders. Using a community-based participatory approach, a purposive sample of palliative care  providers (*n *= 15) in rural areas of Indiana was obtained. Penchansky and Thomas (1981) theoretical framework of access was used to guide the study. A semi-structured individual in-depth interview guide was used to collect the data. All the interviews were conducted online, audio-recorded, and transcribed. Barriers to palliative care  include: misconceptions about palliative care as an underrecognized specialty; lack of trained palliative care providers; late involvement of inpatient palliative care and community hospice services; inadequate palliative care education and training; financial barriers, attitudes and beliefs around PC; and geographical barriers. Facilitators to palliative care include financial gains supporting palliative care growth, enhanced nurses’ role in identifying patients with palliative care needs and creating awareness and informing the community about palliative care. Robust education and awareness, enhancing advanced practice nurses’ roles, increasing funding and resources are essential to improve the access of palliative care services in the rural communities of Indiana.

## Background

Access to palliative care is a universal human right to health, however of the 40 million people globally in need of palliative care, only 14% of people receive it [1] Palliative care is an integrated care approach that supports patients with chronic or life-threatening illnesses and their families by creating awareness and education about the illness and symptom management, providing resources for counseling and bereavement services, and overall promoting quality of life [1]. Individuals with advanced-stage chronic or life-limiting illnesses have complex physical, psychological, social, and existential needs [2, 3]. Ideally, Palliative care should be initiated alongside a diagnosis of a serious chronic or life-threatening illness to address holistic care needs of patients and families, allow adequate care planning, faciltate treatment choices, advance goals of care, and reduce distress among patients and families [4]. Evidence indicate that adequate access and provision of palliative care improves pain and symptom management [5], enables informed decision making [6], reduces hospital readmissions and healthcare costs [7, 8]. Despite multiple benefits, palliative care access remains a challenge in the United States especially in smaller towns and rural communities. This paper aims to highlight various barriers and facilitators in access to palliative care  services in smaller towns and rural communities in Indiana, USA.

### Palliative Care from a Rural Context

Recent health indicators in US, suggest that there is an increased prevalence of chronic illnesses such as heart diseases, cancer, unintentional injury, chronic lower respiratory disease, and stroke [9] among rural residents in comparison to urban population as evidenced by the higher death rates in rural or non-metropolitan areas (39.2%) as compared to urban areas (30.9%) [10]. According to National Review of State Palliative Care Policies and Programs report there is a limited availability of palliative care professionals, resources, and services in smaller towns and rural communities [11]. These geographic inequities are expected to increase with the growing aging population and increased rates of life-limiting illnesses and deaths [12–14].

In the US state of Indiana, there are approximately 6.3 million people are living with at least one chronic disease, and 1.6 million people with 2 or more chronic diseases. Most common are heart conditons(22%) followed by cancer (20.5%) chronic obstructive pulmonary disease (18%), and diabetes (18%) [15]. Majority of these people reside in rural areas and represent the agricultural workforce. Most reported reasons for the high prevalence of chronic illnesses and higher death rates among these people are poorer health, limited socioeconomic resources, restricted access to high-quality emergency and specialty care services including palliative care [10]. The Center for Rural Engagement, Indiana University reported lack of access to health care services as one of the greatest needs of people in rural Indiana. According to them, most people live in counties that are designated as ‘Health Professional Shortage Areas’, where there are only 230.8 active physicians per 100,000 people (compared to the median of the United States of 263.8 physicians per 100,000 people) [16]. Patients in rural communities lack multiple healthcare services including but not limited to post-stroke care, mental health services, complex therapies, hospice services, specialized providers, pain management, however, those services are not found locally and therefore both patients and families experience major healthcare disparities [17]. Medicare beneficiaries also encounter multiple barriers to palliative care access [17]. Limited or no access to palliative care can give rise to further complexities of care, inappropriate treatment choices with increased healthcare risks, caregiving burden, frequent hospitalization, and higher healthcare costs [18]. Integrating and improving access and provision of palliative care should be considered as a major priority area to enhance the quality of healthcare delivered to all Americans especially in rural communities. A strong commitment and engagement are essential in order to improve the access and quality of palliative care services in rural settings, The following paper highlights major barriers and facilitators experienced by the rural communities from the perspectives of healthcare providers and community stakeholders in Indiana, USA.

### Theoretical framework

A theoretical framework of ‘access’ by Penchansky and Thomas [19] was used to guide the study. This model of access has been used widely by several researchers to identify and improve the access and provision of quality healthcare services particularly in both urban and rural contexts of the USA, Canada, and Europe. Penchansky and Thomas define ‘access’ as ‘the degree of fit between the consumer (patient/family) and the service (healthcare system)’. The theory of access incorporates and addresses five specific dimensions that include: accessibility, availability, affordability, acceptability, and accommodation of services [19, 20]. These five dimensions identify a set of specific areas that link the patient and their family and the health care service, essentially a supply-demand relationship, which recognizes both service and user requisites [20]. It is important to note that all these five dimensions are interdependent, addressing one will not significantly improve the access to care unless the other four have not also been addressed [21]. The particular framework served as a comprehensive guide for developing the study tool and provided an analytical framework in identifying perceived barriers and facilitators to access palliative care services in rural Indiana.

## Method

### Study design

 Given the exploratory nature of the study, a descriptive qualitative study using a community-based participatory research approach (CBPR) was designed. A CBPR approach offers a unique collaboration between researchers and community members and allows co-learning and reciprocal transferring of expertise and outcomes [22]. The approach also encourages innovative adaptations of existing resources, explores local knowledge and perceptions, empowers people to act as investigative agents in their own situations, and acknowledges the community as full and equal partners in all phases of the research process, making it an appealing, credible, and useful model for research with vulnerable populations [22]. The qualitative approach in the study recognizes the importance of context, or situation (geographical, physical, and social) in which an experience occurs influences the way people perceive, experience, and interpret the world around them [23], this contextual perspective contributes greatly to perceptions held by rural residents, in this case among the rural residents of Indiana, USA.

### Research setting

 The study was done in Indiana, US. Indiana is located in the Midwestern US, with a total population of 6.8 million in 2020. Indiana has 730 towns and cities where 491 towns are considered as small towns and 72 as rural. The study was done in collaboration with the Indiana Rural Health Association (IRHA), a non-profit agency working to improve the health of residents in Indiana. Other partners include Purdue Nurse Managed Clinics and Purdue Health and Human Sciences (HHS) Extension Educators. There are four nurse-managed and nurse practitioner-led clinics in northern rural counties of Indiana affiliated with Purdue University School of Nursing. Purdue Extension Teams are in 92 rural counties and support social and economic welfare of the rural communities. The study was funded by the AgSEED short for Agricultural Science and Extension for Economic Development, Purdue in April 2020, however, the study got delayed for a year due to the COVID-19 pandemic. Data collection began in Jan-April 2021. Ethical approval for the study was obtained from the Institutional Review Board, Purdue University, West Lafayette, IN, USA. The study followed the revised ethical guidelines as provided by the University during the pandemic.

### Participant recruitment and sampling

 Participant recruitment was done with the assistance of community partners as listed above. Online flyers and posters containing the study information sent through emails, and social media using the Facebook page of collaborative partners in the study. The snowball approach was also used for recruiting eligible participants. The participants approached the research team using emails and phone calls. The study information and consent form were sent online to the eligible participants along with the preferred timing for scheduling the interviews. A purposive sample of rural healthcare providers and community stakeholders (***n ***= 15)was obtained. Healthcare providers included physicians, nurse practitioners/clinical nurse specialist, registered nurses, case managers, social workers, and chaplains, whereas stakeholders were defined as managers and administrators of different homecare/hospice agencies, clinics, and hospitals. All the participants were 18 years and older and experienced providing generic or specialized palliative care services to the patients and families residing in the rural communities of Indiana, USA. Participants were recruited in assistance with the IRHA, Purdue Ag Extension, and Purdue Nurse Managed Clinics in Indiana.

### Data collection and analysis

 Data was collected using semi structured interview guide. The interview guide was developed using Penchansky & Thomas [19] framework and relevant palliative care literature and was finalized after having an expert opinion from IRHA and palliative care experts from the university. The five specific domains of access such as accessibility, availability, affordability, acceptability, and accommodation of services described in the framework is added in the interview tool. Addiitonal questions were added including awareness, cultural and psychosocial concerns, and transitions in care. Semi structured interview guide allows an open dialogue between the researcher and the participants, often supplemented by follow-up questions, probes and comments. Using this flexible interview tool, the researcher is able to collect rich and in-depth meaningful data while exploring participants’ thoughts, feelings, expreinces, and beliefs about a particular topic under study [24]. Interview guide is provided in Table 1.

**Table 1:**
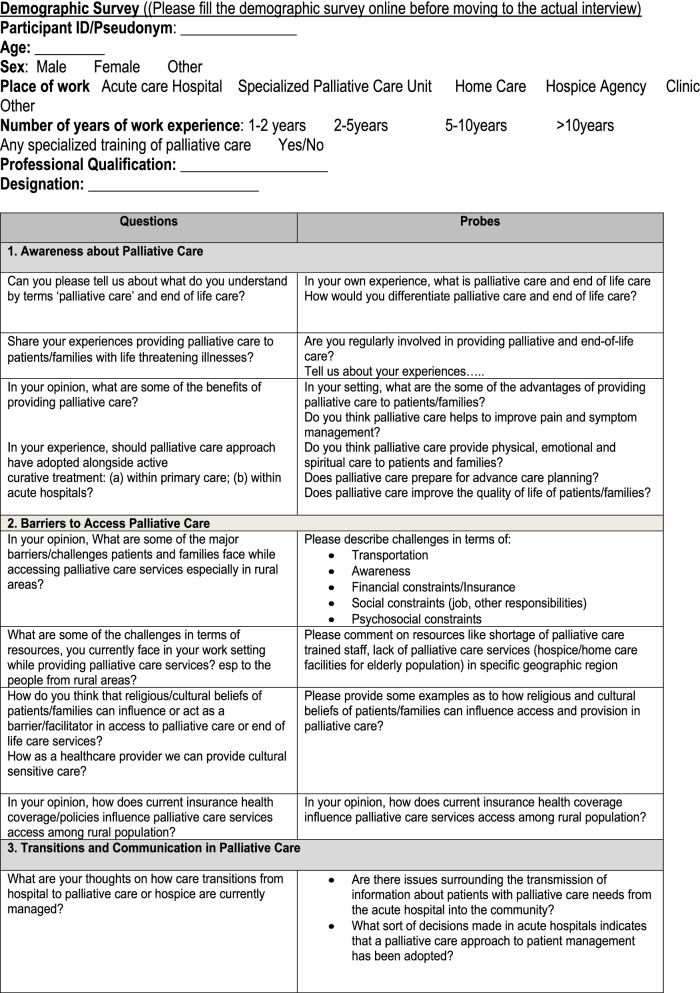
Interview guide

Interviews were conducted by the PI along with a doctoral student. PI is primarily a qualitative researcher and has done multiple qualitative interviews before in her previous studies. During the initial five interviews, PI sat with the doctoral student during the inteviews, facilitated the interviews and provided the training and advice to the student to conduct the future interviews. Once the student felt comfortable, rest of the interviews were conducted by the student herself. All the fifteen interviews were done online using Zoom or Microsoft Teams as preferred by the participants. Before each online interview, participants were explained about the study, asked to provide verbal consent along with permission to record the interviews. Each interview was about 30-60 min on average. Interviews included asking participants about the demographic information as well as their experiences and available palliative care  services in the area. All the interviews were recorded, transcribed, and stored in the institutional online data repositoryr after removing all the personal identifiers. A thematic analysis approach was undertaken to analyse the data using a constructvist interpretive paradigm [25]. The particular paradigm assist the researchers to understand and interpret the underlying meanings in the data in order to better understand the phenomena under study. The data analysis process involved inductive reasoning, constant engagement, testing, challenging preliminary interpretations and, finally, conceptualizing to understand the meaning, concepts, and patterns generating in the data. All the recorded data was transcribed, read, coded, and put into separate categories by a team of 3 researchers including PI along with two doctoral students. Each category formulated was defined, discussed among the team members and were put under separate themes. Regular meetings were held among the team members to discuss the codes and categories emerging in the data as well as to process continuous reflection, ongoing critical questioning and coherent reasoning to avoid any premature closure or overdetermination of the patterns and meanings in the data [25]. An iterative process of analysis was undertaken, emerging categories, linkages, patterns, and themes in the data were shared among the research team members and a final thematic list was generated with consensus among the team members  [25]. To ensure trustworthiness and and rigor, a separate audit trail was kept about all the research activities. Separate logbooks, field notes, and reflexive notes were also maintained throughout the study.

## Findings

Demographic profiles of the 15 participants include age, gender, job title, facility type, years of experience in palliative care and palliative care training experience. The demographic findings are given in Table 2.

**Table 2:** Participant profiles (*N* = 15)

	n	%
Age		
25-35	7	47
36-45	3	20
>45	5	33
Gender		
Female	13	87
Male	2	13
Job title		
Registered Nurse (RN)	7	47
Nurse Practitioner (NP)/Clinical Nurse Specialist (CNS)	3	20
Palliative Care Physician (MD)	2	13
Social Worker (SW)	2	13
Chaplain	1	7
Facility type		
Hospital	9	60
Hospice	3	20
Primary care clinic	2	13
Nursing home	1	7
Palliative care experience (Years)		
1-2	1	7
3-5	8	53
6-10	3	30
>10	3	20
Palliative care training		
Yes	9	60
No	6	40

Findings obtained from the interviews are categorized under various themes. The themes reflect barriers and facilitators to palliative care access among patients and families in the rural communities. Seven themes describing barriers to palliative care access emerged as: (1) misconceptions about palliative care as an underrecognized specialty; (2) lack of trained palliative care providers; (3) late involvement of inpatient palliative care and community hospice services; (4) inadequate palliative care training for clinicians; (5) financial barriers to palliative care development; (6) cultural barriers and (7) geographical barriers. Three themes describing facilitators to palliative care emerged as: (1) hospital administration supporting palliative care growth because of financial benefits; (2) hospital nurses helping identify patients with palliative care needs; (3) word-of-mouth recommendations of hospice services in the community.

### Barriers to Palliative Care Access

#### Misconceptions about palliative care as an underrecognized specialty

The lack of a clear and well-disseminated definition of palliative care as a specialty impedes its recognition and understanding among healthcare professionals. Participants had multiple concerns regarding the clear definition and regulation of palliative care services:

*“So typically, the timeframe that people really look at palliative care is two years. People with a two-year survival from their illness … two years or less … I don’t think it’s official [definition], but that’s sort of a commonly accepted [definition].” (MD2)*.

*“…defining palliative care [is important] …just making it distinct from hospice.” (RN1)*.

*“I have been concerned in the past about hospice agencies and other health care systems kind of terming a program palliative care when it’s really not at all.” (NP1)*.

Participants also verbalized not having clear guidelines for practicing palliative care  in outpatient and community practice settings as compared to inpatient areas leading to confusion, misunderstanding, and delay in providing adequate palliative care services.

* “I think there are standardized guidelines when it comes to an inpatient palliative care team. I don’t think there’s much guideline at all when we’re talking about palliative care  in an outpatient or in the community.” (NP1)*.

Often palliative care is understood as *‘giving up on you’ or ‘it’s the end of life*’ which makes it further difficult for the clinicians to communicate about or make referrals for palliative care services. Such an understanding is common among the clinicians as well as the general public.

*“Oncologists still have this idea that If I tell people I’m gonna send you to palliative care, I’m giving up on you and I’m not going to tell my patient.” (MD1)*.

*“We [inpatient palliative care team] get barriers in that we tend to deal with other physicians sending mixed messages [about palliative care] to patients.” (MD2)*.

Similarly, patients and families often equal palliative care with hospice care.

*“And people think it [palliative care]’s the same thing as hospice, you know, so those words don’t mean different things to people” (RN2)*.

Other than lack of understanding and clarity about the palliative care, another major barrier is the lack of trained palliative care providers in the area, another theme in the study.

### Lack of trained palliative care providers

Most participants shared that there are not enough trained or specialized palliative care providers. In inpatient settings, there are only a small number of specialists, including both physicians and advanced practice nurses. First of all, palliative care is not a well-paid specialty:

*“It’s not a field that pays that well, you know there’s a lot more ways to make a lot more money.” (MD2)*.

Also, some palliative care providers may only practice part-time:

*“There was one provider at X [a local hospital] who was an oncologist. He really only did inpatient palliative care 25% of the time, which was ridiculous, like you got a bigger hospital than we do…” (MD1)*.

Despite rising demands and multiple benefits of palliative care, it was sad to note that some hospitals had downsized their palliative care team and were planning to close these services in the near future.

*“Hospital decided to downsize the palliative care team, so now they’re no longer in house. They are more with the oncology group and mostly outpatient.” (RN1)*.

Participants conveyed that there lacks outpatient palliative care in the community and therefore, the quality of care suffers. They feel limited in providing comfort care, pain management services, adequate consultation, and guidance to improve the quality of life of their patients and families.

*“We don’t have a palliative care team [in primary care clinic], and I mean actually palliative care. It’s something that’s come up quite a bit in our discussions in our meetings because quite a few of us are now getting palliative care patients all of a sudden, and how do we provide the best quality care? How do we assess where they are?” (SW1)*.

*“We don’t really have anything at all in our eight county area for outpatient services.” (MD1)*.

Following up on patients residing in rural and remote areas after their discharge was an additional concern among the service providers.

*“It sometimes is difficult to try to make sure you can get follow up with those people [after hospital discharge] and sometimes one of the reasons we [inpatient palliative care team] end up following them is because I can’t really get anybody to write the opiates [in the community].”(MD1)*.

Participants also added that most resources are available and limited to hospice agencies only.

*“In some areas and some counties in Indiana, less hospice agencies even go to those areas [remote or rural areas]. What a lot of people don’t understand is that out in the community there’s really not palliative care resources outside of Hospice. ” (NP1)*.

Lack of resources and trained providers in the community often resulted in delayed care and lengthy care transition process, another major barrier in the adequate access to palliative care in rural communities.

### Late involvement of inpatient palliative care and community hospice services

Participants shared that both inpatient palliative care consultations and community hospice services are introduced too late as the disease progresses which causes additional sufferings for the patients and families managing illness, decision making, and coping with additional care responsibilities.

*“I feel like [inpatient] palliative care is brought in a little too late. We find that the consultancies are happening when the patient is already to a point where it looks like they’re going to pass sooner than later.” (RN1)*.

*“They [patients]only get to us [Hospice] when it is the only option.” (RN2)*.

*“Still, because people [clinicians and patients] don’t get the idea that palliative care should begin way upstream, you know it should begin literally at the time of or shortly after diagnosis of a life limiting illness.”(MD1)*.

Participants also reported that clinicians’ training background, self-attitudes, and practice philosophy may impede the early involvement of palliative care for patients with life-limiting illnesses and therefore, needs to be considered a high priority for improving the palliative care services in the area.

*“I think for a lot of doctors. It’s a little tiny bit of a pride thing. Hospice means they lose their patient.” (RN2)*.

*“Especially for physicians and surgeons who can take really aggressive measures to treat a patient. They know all of the advanced treatments and surgeries and technology and that they want to exhaust all of their actions before considering getting palliative care involved.” (RN3)*.

Training is essential in the provision of adequate palliative care in the community, discussed as another theme in the study.

### Inadequate palliative care education and training

Many of the participants acknowledged that palliative care education/training for clinicians in all care settings is inadequate and poses major communication and illness management barriers for the rural communities.

*“Palliative care wasn’t a thing when they [physicians] were training, so they never knew they never had it. They never used it; they never knew how to incorporate it into their practice.” (MD1)*.

*“I only had like two nurse practitioners when we first started. But during that time, you would think that people would get to know what palliative care does, and yet for the vast majority of my colleagues, It doesn’t seem to resonate with them and some of that is sort of cultural within their specialties.”(MD1)*.

*“Palliative care, I’m not too sure because that to me is actually been a new term, especially this last year I’ve been learning about it, so I feel like our services [in the nursing home] for palliative care probably not as great as Hospice care, since I feel like we’ve had known about Hospice care for a long time.” “Even our staff they probably really don’t know the difference between palliative and Hospice care.” (RN4)*.

Participants indicated that there is no requirement for continuing education specifically in palliative care for clinicians other than palliative care specialties:

*“I don’t think anyone’s required to have continuing education hours on palliative care.” “I don’t find my hospital pushing for it [palliative care continuing education]” (RN1)*.

Consequently, most clinicians lack confidence and are often not comfortable talking about uncertainties, psychosocial and spiritual concerns, advanced care planning, pain management and comfort care treatment options available in the community with the patient and families.

*“We have had our nurses talk to the families about it over the phone, but I think depending on the nurse and how much experience they have communicating with the family on hospice care, a lot of the families come at the nurses and are like just getting so angry at them.” (RN4)*.

* “Why we [inpatient palliative care team] get involved is that the nurse has been talking to the patient and they’ll start bringing things up, and they’ll be like ‘I can’t talk about that [palliative care] with them’.” (MD1)*.

* “I have had some primary care providers call me [palliative care specialist] and say, hey, here’s this patient. Sometimes they just want to ask me questions. You know, I think they need more support with this.” (MD1)*.

Participants reported that often it is difficult for them to communicate or respond to unique palliative care concerns and cultural needs, especially among specific minority rural communities.

*“Every time I see the palliative conversations take place with the Amish specifically, I feel like it’s like a brand-new concept. I’m not sure in their culture what palliative care means to them.” (RN1)*.

It was also interesting to note that spiritual or existential care i.e. an important aspect of palliative care philosophy  is often relegated to chaplains in the care settings. For most nurses and doctors, attending to spiritual and existential concerns is the responsibility of chaplains or faith healers. For some, due to time constraints and workload, spirituality is often given less priority among the other domains of palliative care.

*“It’s a very valuable thing to have dedicated spiritual care and social work. Because they both can have much more fruitful conversations with families, more so than I [palliative care physician] and my NPs can. We’re still…though we may be a little more enlightened. My skill set is still not there. With either chaplaincy or the social work part of it, so I’m mostly medicine to begin with.” (MD1)*.

### Financial barriers

Among other barriers, financial barriers were also reported impeding palliative care access and provision among communities. Participants highlighted that finding financial support for palliative care development can be challenging:

*“ ‘We [hospital administration]’ll keep you [palliative care team]around now’ but it doesn’t mean that they’re willing to fund anything out of the ordinary that you still have to go to him and say, OK, I have to beg and beg and beg and beg and beg, then we have another nurse practitioner.” (MD1)*.

*“Really our [palliative care team] mission is the same as everybody else. They [hospital administration] want to make money so everything that you present to the administration has to have the financial component front.” (MD1)*.

*“I know that X [a local hospital] had an outpatient clinic for a period of time but could not continue to support it from a staffing perspective.” (NP1)*.

*“We [nursing clinics] don’t have the resources to provide it [palliative care] … I haven’t looked a lot to see what HRSA (Health Resources and Services Administration) has, but I don’t think it’s one of their priorities right now for funding. …the funding base needs to be there… we [nursing clinics] would have to be able to apply for funding or something.” (NP2)*.

Participants conveyed that financial constraints can be a barrier to palliative care access among patients and families, especially those who are not eligible for Medicare:

*“I think definitely the financial piece of it is a huge concern also for families. You know whoever has a life ending illness ends up on disability and then the partner can’t work, and the family circles financially. That’s a major concern.” (SW1)*.

*“Especially like some of our (primary care) clinics (in rural areas) where we have that high Hispanic population where they are our self-pay (patients) and don’t really have any insurance or anything like that to be able to afford it [palliative care].” (NP2)*.

Funding and resources were found essential to continue and support palliative care training and services among providers and therefore, need special consideration at both systems and policy level. Besides financial and other barriers, participants also reported several contextual and cultural barriers in accessing PC services which are reported as the following theme in the study.

### Attitudes and beliefs around palliative care

Participants perceived that often patients’ and families’ own attitudes, values and beliefs around palliative care serve as a barrier to its access and provision.

*“People who are struggling with their diagnosis [would not want palliative care]. You know they don’t really like the idea that they have a life limiting illness and that’s what we [palliative care team] talk about.” (MD2)*.

*“It [palliative care] is a discussion that is still very uncomfortable for a lot of my nurses. I think a lot of it too is it’s uncomfortable at a level of even the patients just refused to hear about it, so there is a lot of them that are in denial” (RN7)*.

Patients and families deny or lack a clear understanding of palliative care or are unable to accept such care for their family member suffering from advanced illness due to fear, familial and cultural roles and expectations, values and beliefs about life and death.

*“When a physician initiates it, it’s just that fear from the parent. it is pushed back in there. They shut it out, you know like ‘no, I don’t want to hear that’, and I think sometimes it can make the medical team a little uncomfortable.” (RN5)*.

*“People think that a miracle is going to happen, and which is not going to, so they kind of disregard the discussion regarding how serious it is with their loved ones.” (NP1)*.

It is significant to note that most patients and families residing in the rural communities in Indiana belong to farmers’ communities. Farmers’ healthcare beliefs including palliative care delays were highlighted by participants.

*“I find that a lot of the patients that are farmers. They usually don’t seek medical treatment until it’s like absolutely necessary, like last case resort. I feel like palliative care is more proactive and you should be getting involved super early. It’s more of a preventative thing in my eyes and I don’t know if that’s something they prioritize in their health care.” (RN1)*.

*“A lot of people from rural populations on farms or you know they maybe work in their town and so they might not take the time to be seen until it’s truly an emergency and there have been patients who got chest pain for months and are just getting around to getting it looked at.” (RN3)*.

Farmer families may have a better acceptance of death and thus don’t perceive the need for palliative care.

*“I think farm people in general are a lot more accepting of death and a lot less afraid of death. I think they just get it. It’s inherently part of life on the farm that you have life on the farm but there’s also death on the farm.” (MD1)*.

### Geographical barriers

The geographic distance can be a barrier to access. Long travel to appointments is especially challenging for patients in poor health conditions.

*“One of the last guys I [palliative care physician] saw as a home visit was a gentleman who was getting transfused all the time. He had leukemia and a different kind of cancer and he had heart failure, whole bunch of stuff and he was in a wheelchair and he had chronic swelling in his legs and it was just a big ordeal every time his wife had to come and bring him in to do anything.” (MD1)*.

*“Location is another huge barrier because if they have to go into you know into Lafayette or into Indy or whatever a lot of them don’t have that transportation.” (NP2)*.

For home-based services, hospice agencies often have distance limits that professionals may not be able to offer home visits to remote areas. This further gets complicated during harsh weather situations.

*“[Long drives can be a barrier to access], of course, if they [patients] are very remote. Our Hospice tries to make sure we can always get to a patient’s home within an hour, so obviously we’re not a rapid response team.” (Chaplain)*.

Other than multiple barriers, the study also found out certain facilitators to improve the access and provision of palliative care services. These themes are as follows.

### Facilitators to palliative care access

#### Financial gains supporting palliative care growth

Few participants believed that the provision of inpatient palliative care has resulted in reduced length of stay, repeated hospitalization, and cost-effectiveness, all of which are considered as key reasons why palliative care has received a lot of administration support in recent days. This has served as a major facilitator in upbringing the support for palliative care.

*“We [inpatient palliative care team]’re following up after we’ve seen them in the hospital is because we’re more worried about them getting re-admitted and all of those kinds of things.” (NP1)*.

*“We have huge savings in length of stay, so you identify early on what people goals of care are and what’s a you know the best thing for their care. You can keep them out of the ICU’s, and you can keep him from having expensive procedures, so the costs are down, the length of stay is down.” (MD1)*.

Other than financial gains, nurses’ role recognition in palliative care was another major facilitator found in the study as follows.

#### Advanced nursing practice roles in identifying patients with palliative care needs

Participants felt that bedside nurses as well as nurse practitioners in rural clinics can play a key role in facilitating and supporting palliative care. Nurse practitioners in rural outpatients clinics should be informed about adequate resources and training tools. These tools may help nurses to identify and communicate timely about patients who can benefit from palliative care. Advanced nurse practice roles could promote early goals of care planning, decision making, and proper illness management especially in low resourced rural areas.

*“We had a little like pocket card. That are the things that would make this patient appropriate for palliative care, and we hand those out to the nurses to try to get them used to. Hey, if you’re seeing somebody that has multiple comorbidities, they’ve been re-admitted many times or within the last 30 days or whatever, they have complex family dynamics, they have psychosocial issues, they have uncontrolled symptoms. All these things were on the card. Please call palliative care.” “Now we have a pretty, I’d say, well informed nursing staff that works very closely with us, and often they’re the facilitator for getting us consults.” (MD1)*.

*“My role is mainly trying to get palliative care on board and suggest to our primary physicians. Can we consult the palliative team to just talk to family about goals of care?” (RN1)*.

Nurses as primary care providers can play an integral role in early palliative  consultations resulting in better coordinated and effective holistic care.

#### Creating awareness and informing the community about palliative care

Participants felt that often people in rural communities are not aware of the services available in the area or sometimes services are located far from their own reach. As a result, they experience difficulties in managing care, go through additional burdens and stress.

 Healthcare providers conveyed that the hospice agents they work for have well-established multidisciplinary hospice teams and great service coverage, especially for Medicare beneficiaries. In the communities, good experiences with hospice spread within social networks that facilitate the usage of hospice services:

*“We [hospice] have a team. Every patient has a chaplain, social worker, nurse, home, health aide. We have a music therapist also, so the direct questions and discussions about their spiritual concerns.” “We’ve had families that need food. Need help with food and we can do some. You know some small grocery gift cards or whatever but as far as what the patients need directly. It’s pretty much all supplied, I mean under pads for the bed, diapers, bathing stuff.” (RN2)*.

*“It [hospice]’s word of mouth and if people have had a good hospice experience, or a bad one, unfortunately, but it travels, 10 to 20. Maybe at their funeral they talk about how great it was. Or you know, whatever, and I think the rural population is actually pretty aware of hospice now.” (Chaplain)*.

## Discussion

Study findings provide insights into several barriers and facilitators to palliative care access from the perspectives of providers who serve rural Indiana. To the authors’ knowledge, this is the first study looking into palliative care access in rural Indiana. Within the context of inpatient palliative care, findings are evident that barriers to palliative care access among rural populations were critical. Palliative care, as a newer specialty that comprises end-of-life care or hospice, has not gained adequate familiarity among both clinicians and the public in rural Indiana. However, community hospice care has gained more familiarity as it has been long-standing and well covered under Medicare.

The findings in this study highlighted that misconceptions about inpatient palliative care such as equaling it to end-of-life care or hospice were common among clinicians and patients and families. Similar results were found in our recent scoping review where multiple studies pointed out to the fact that healthcare practitioners including primary care providers, nurses and practitioners either lack the general understanding and clarity around palliative care concepts [26]. Despite the recent development in palliative care awareness, education and training, providers remain unable to recognize the needs and significance of it  which further results in delayed care for the patients and families. There could be several reasons for this emerging argument. Often the reason for the inadequate provision of palliative care  is not limited to only the knowledge and awareness of providers, it is how the service is perceived or practiced in the facility. The organizational climate and culture are not unable to support the ability of providers for adequate palliative care referral and consultations in the facility. Providers’ own values and belief system, lack of continuing education, and not having the necessary tools or education to recognize the needs and requirements of chronically ill patients and their families are often additional barriers in the provision of palliative care. Multiple definitions and regulatory guidelines around palliative care cause further confusion and lack of clarity towards its better understanding in care practices among providers [27].

The lack of community-based palliative care  approach found in our study aligned with the findings of previous researches [28–31]. Inpatient services with minimal community outreach can be a critical barrier to care access as this approach excludes the vast majority of people living with life limiting illnesses in the community. Within nurse managed  primary care clinics in rural communities, providing palliative care  was found to be an overstretch of practice scope due to the lack of funding support. Even the existing inpatient services were involved too late, mostly towards the end of life. Providers’ training background and practice philosophy and the culture of specialty areas (e.g., oncology) can vary a lot that root their exposure to and beliefs in palliative care. Inadequate palliative care training for all clinicians was  fundamental to provider misconceptions and late or no referrals to palliative care for patients with life-limiting illnesses.

Our findings also indicated that the lack of acceptance of palliative    is associated with the fear and denial   among patients and families. Palliative care  is often viewed as ‘*giving up*’ or ‘*end of life*’ as evident in the findings. Such cultural meanings and values associated with palliative care  were also major barriers towards appropriate communication, referrals, and consultation. Literature also supports that in rural communities, there are multiple contextual barriers impeding PC services including occupations and regional and generational cultures [30, 32, 33]. From the rural Indiana context, for example, a lot of people are farmers who commonly play down preventive health services. Death and dying may be well accepted spiritually among these farming communities but the benefits of palliative care beyond the end of lives need to be further disseminated.

Moreover, the geographic location, lack of transportation, and financial constraints were found to be other barriers in accessing palliative care, especially in rural communities. The nature of work and employment, healthcare facilities located far from home, limited availability of transport esp. during winters or harsh weather conditions often create additional challenges for the people in the rural areas to access services [30, 31, 33, 34]. In addition to the limited availability of palliative care  providers and facilities in rural areas shown in other similar studies [35–37], our study highlighted the challenging atmosphere for palliative care to grow in hospitals as a not well-paid specialty and fewer providers choose to practice palliative care. Furthermore, some facilities were downscaling the available palliative services and resources in rural areas due to a lack of funding. It is also important to note that this study was done during the second phase of the Covid pandemic, this was a significant time when palliative care service was highly needed among the patients and families. Lack of palliative care availability during these times could have further added to healthcare disparities and sufferings among the underserved rural communities in the area.

Apart from previous studies, our study highlighted some novel perspectives around facilitators of palliative care  including strengthening advanced practice nurses’ roles to improve palliative care  access and utilization in the rural communities. Findings are evident that rural clinics run by nurse practitioners were providing referrals for the palliative care  consults in the areas. Literature is evident that nurse practitioners are cost-effective, resourceful, and found to be effective in responding to the primary care needs esp. in rural and underserved communities [38]. Advanced practice nurses can play a key role in reducing disparities and improving the community outreach. Findings also supported that palliative care trigger tools were found to be useful among nurses to recognize palliative care needs and consultations for patients and families, especially in inpatient settings. More robust palliative care  training and education and additional incentives to participate in these educational training sessions were also highlighted as major facilitators of PC in healthcare and community settings. Few providers in the study did perceived financial gains or economic benefits as a facilitator of palliative care in the study however given the limited scope of the study, further studies are suggested to examine in detail about the economic or financial benefits of PC provision in rual settings.

### Strengths and limitations of the study

The study is first of its kind among rural communities of Indiana and  provides an enriched understanding of palliative care  access and delivery from a diverse perspectives of healthcare providers including nurses, nurse practitioners, social corkers, chaplains and nurse managers from both inpatient and outpatient settings. The rich descriptions generated  may help us to critically understand and identify ways to improve the palliative care delivery in the rural communities. Limitations include non generalizability of the findings as the findings are limited to rural Indiana context. Moreover, there is a possibility of social desirable responses of the participants in the study. At some instances, we faced internet connectivity issues while doing the interviews and had to reschedule some interviews at different times. It is possible that some participants may not be comfortable using the online medium for interviews that may have possibly influenced findings in the study.

#### Implications

Palliative care is a human right, recent studies have shown its multiple benefits from specific health, social, and economic perspectives [39–42]. Especially in rural communities, access to palliative care requires a lot of attention to reduce the healthcare burden and prevent disparities. Our study strongly suggests the need to increase the awareness of palliative care among both users and providers at education, policy, and practice levels. Healthcare policies need to put more emphasis on the utility and provision of palliative care. Efforts need to be in place for creating a clear definition of palliative care.   Advanced practice nurses’ placement in rural regions can further enhance the availability and access of palliative care in rural communities. Proper policy guidelines and regulatory mechanisms tailored to the cultural and contextual needs of the rural population needs to be in place and emphasized among the healthcare community. Future funding support is needed to enhance community outreach services and resources to improve palliative care  access and availability in rural areas.

## Conclusions

Lack of palliative care  access can be a major factor affecting the healthcare conditions in rural and underserved communities, overall influencing rural growth and wellbeing. Further emphasis and efforts are needed at both practice and policy levels to enhance the awareness, importance, and availability of adequate palliative care  services in rural communities. Strengthening advanced practice nurses’roles and appropriate allocation of funding and reosurces can  create new pathways for ensuring accessible palliative care services in the  rural communities of Indiana.

## Data Availability

The datasets used and/or analysed during the current study is available from the corresponding author on a reasonable request.
